# Initial Co‐Design Development of a Questionnaire to Measure Patient Preferences in a Danish Mental Healthcare Setting

**DOI:** 10.1111/hex.70561

**Published:** 2026-01-15

**Authors:** Klaudia Kristensen, Anna Skov, Solvejg Kristensen, Ottar Ness, Søren Paaske Johnsen, Mainz Jan, Malene Terp

**Affiliations:** ^1^ Psychiatry North Denmark Region Aalborg Denmark; ^2^ Danish Center for Health Services Research, Department of Clinical Medicine Aalborg University Aalborg Denmark; ^3^ Norwegian University of Science and Technology Trondheim Norway; ^4^ Department of Health Economics University of Southern Denmark Odense Denmark

## Abstract

**Background:**

Despite decades of quality improvement efforts in mental healthcare, patient preferences remain insufficiently integrated into care planning and system design. Although person‐centered care strives to align services with patients' most valued preferences, current tools for measuring those preferences often fall short. Embedding co‐design approaches—where patients and other stakeholders collaborate as equal partners in tool development—is essential to ensure that measurement instruments are both relevant and resonant with lived experience. This study aimed to develop a questionnaire that captures patient preferences in mental healthcare through a co‐design process that actively integrates lived experience, thereby enhancing face and content validity.

**Methods:**

The development of the questionnaire followed four key phases: identification of domain, item generation, content validity, and pre‐testing of questions. A co‐design approach was integral to the entire process, with active collaboration with the co‐researcher or co‐designers at every phase.

**Results:**

Nine themes were identified in the literature, and two novel themes, “the person as a whole” and “meaningful community connections” emerged through co‐design and extend beyond prior studies. From 242 preliminary items, workshops refined the questionnaire to 58, and content validation further reduced it to 54 items with recommendations for clarity, simplicity, and anonymous responses. Pre‐testing of questions confirmed the questionnaire's usability and face validity.

**Conclusions:**

This study demonstrates how traditional questionnaire development can be supported by co‐design to effectively develop a patient preference questionnaire that is both valid and meaningful within the Danish mental healthcare context. While limitations exist, particularly regarding group dynamics and representation, the process sets a strong foundation for future work.

**Patient or Public Contribution:**

This study was co‐designed in collaboration with individuals with lived experience of mental illness. A co‐researcher with lived experience was involved throughout several stages of the research process, including the design and facilitation of workshops, recruitment of participants, and interpretation of findings. Additional 15 co‐designers with lived experience were engaged in two workshops, contributing actively to the generation, refinement, and validation of questionnaire items. Their insights ensured that the tool reflects the values and preferences of those it aims to serve. Peer workers, clinicians, and researchers also supported the co‐design process, helping facilitate workshops and integrate experiential and academic perspectives.

## 1 Introduction

Over the past decades, considerable efforts have been dedicated to enhancing the quality of mental healthcare systems [1, 2]. Central to this improvement is the growing emphasis on person‐centered care, which among other things places patient preferences at the heart of care planning, organization, and content development [3, 4, 5]. However, no validated gold‐standard measure specifically captures patient preferences in mental healthcare. Existing tools often measure preferences only indirectly, lack comprehensive domain coverage, or are not fully validated psychometrically [6, 7, 8].

What patients deem most important in person‐centered care is not always adequately captured due to several challenges in policymaking, clinical practice, and research. First, our recent scoping review, which aimed to identify essential components for patients when interacting with the mental healthcare system, revealed several gaps [9]. There is no systematic and uniform method to assess patient preferences, and existing questionnaires measuring preferences in mental healthcare require updates. Second, clinical practice often focuses on patient satisfaction, but while mental healthcare patients frequently report high satisfaction levels, current surveys may fail to capture critical aspects of their needs and preferences [10]. Lastly, policymakers tend to prioritize specific quality measures and targets, which can be difficult to assess and may not align with what patients consider most important [11].

Data to measure quality of care from patients is often gathered using questionnaires. In the traditional questionnaire development process, several key phases are generally followed to ensure the tool's reliability and validity, including identification of domain, item generation, content validation, pre‐testing, and psychometric validation [12]. Two important aspects of this process are face and content validity. Face validity refers to how clear, relevant, appropriate, and sensitive the questionnaire appears to its intended audience. Content validity, on the other hand, concerns whether the questionnaire adequately captures the full scope of the concept it is intended to measure [13, 14]. The standardized process of questionnaire development is essential, but often only involves patients in part of the process. Engaging patients meaningfully in survey design such as in the development of an arthritis intake experience survey is shown to enhance the relevance, clarity, and utility of the developed tools [15]. However, this is rarely done. None of the studies in our scoping review involved patients as active collaborators throughout the full questionnaire development process [9]. When patients are not engaged throughout the entire process, there is a risk that the developed tools fail to reflect true preferences, use inaccessible language, or miss critical aspects of person‐centered care [16]. Thus, this underlies a possibility to enhance the quality of designing the health care system truthfully based on what is important for patients.

To address this gap, patients' voices must not only be heard but actively integrated into the development process. Co‐design offers an inclusive and collaborative approach that directly responds to the lack of meaningful patient involvement in traditional methods [17]. At its core, co‐design is defined by active partnerships, where patients, staff and researchers work together as equal collaborators to understand problems and jointly design, develop, implement, and evaluate innovative solutions [18, 19]. Rather than being consulted at isolated stages, patients in co‐design processes contribute with lived experience and insight throughout every phase, ensuring that the resulting tool is relevant and thereby can minimize response error and burden [17].

This paper details the initial development of a questionnaire designed to capture patient preferences in a Danish mental healthcare setting, using a co‐design approach to ensure face and content validity. It outlines the iterative co‐design process used to develop the questionnaire and presents the resulting instrument. Thus, the aim of this study was to describe the initial development steps of the development of a questionnaire to capture patient preferences in a Danish mental healthcare setting. Specifically, we explored how co‐design contributed to enhancing the relevance and comprehensiveness of the resulting instrument in four phases: (I) identification of domain, (II) item generation, (III) content validity, and (IIII) pre‐testing of questions.

## 2 Setting, Methods and Material

### 2.1 Setting

This study was conducted within the Danish mental healthcare context. The activities of the study took place at the Psychiatry, Region North Denmark, with affiliations to Center for Recovery and Co‐creation and Danish Center for Health Service Research. All activities were carried out between March 2022 and May 2023.

The activities described in Table 1 were carried out by the primary researcher in collaboration with a co‐researcher. The co‐researcher, a former psychiatric patient, was at the time of the study a bachelor student completing an internship at Center for Recovery and Co‐Creation. Her role as a co‐researcher, was defined as a collaborator with lived experience, who played an active role throughout multiple stages of the research process. This included planning workshops, recruiting participants, co‐facilitating workshops, providing ongoing feedback, and contributing to the interpretation of findings.

**Table 1:** Overview of the process with identified phases, activities and participants.

Phase	Activities	Participants
1. Identification of domain and conceptualizing	1. Reviewing literature to identify key themes.	1. Primary researcher
	2. Recruitment of participants for item generation workshops.	2. Co‐researcher and primary researcher
	3. Strategic planning of workshops in item generation phase.	3. Co‐researcher and primary researcher
2. Item generation	4. Workshop 1: Mapping key aspects of care pathways and prioritizing themes via color‐coded ranking. (Discover and Define)	4. 15 co‐designers: Seven current patients (three males, four females, aged 18–45). Three former patients (all female, aged 41–45). Five peer workers (three males, two females 32–42).
		Three facilitators from Center for Recovery and Co‐Creation. Primary researcher, co‐researcher.
	5. Identification of relevant items in literature. (Develop)	5. Primary researcher and co‐researcher
	6. Workshop 2: Prioritizing and refining questionnaire items (Deliver)	6. 15 co‐designers: Seven current patients (three males, four females, aged 18–45). Three former patients (all female, aged 41‐45). Five peer workers (three males, two females 32‐42).
		Three facilitators from Center for Recovery and Co‐Creation. Primary researcher, co‐researcher.
3. Content validity	1. Structuring items in Research Electronic Data Capture (Redcap) a secure, web‐based tool for building and managing online surveys and databases in research. (Deliver)	1. Primary researcher and a statistician.
	2. Removing item duplicates and refining language. (Deliver)	2. Co‐researcher, primary researcher, a co‐designer, a project nurse, and a statistician.
	3. Discussing response categories and overall questionnaire design. (Deliver)	3. Co‐researcher, primary researcher, a co‐designer, a project nurse, and a statistician
4. Pre‐testing questions	1. Pre‐testing of questions conducted using “thinking aloud” method to assess clarity, usability, and design.	1. Primary researcher and 13 co‐designers (three males, ten females, all from the municipality of Aalborg, all white, in out‐patient treatment)
	Iterative adjustments made until no further changes were needed.	

The primary researcher led the overall study, coordinated the project, continuously consulted relevant literature throughout the process to ensure methodological coherence and consulted the other authors when needed.

The other participants in the study, referred to as co‐designers, included a diverse range of adults with lived experience of mental illness. They were either current patients, former patients or peer‐workers. They were recruited through two main channels: the Peer Board, a user council affiliated with Psychiatry, Region North Denmark and through peer workers employed at clinical departments. Peer workers are individuals with lived experience who are employed to support others in their recovery journeys [20] and in service development [21].

In addition to the co‐designers, a group of project collaborators including researchers, facilitators, and clinicians affiliated with Psychiatry, Region North Denmark contributed to the study.

### 2.2 Overall Methodology

**Figure 1:**
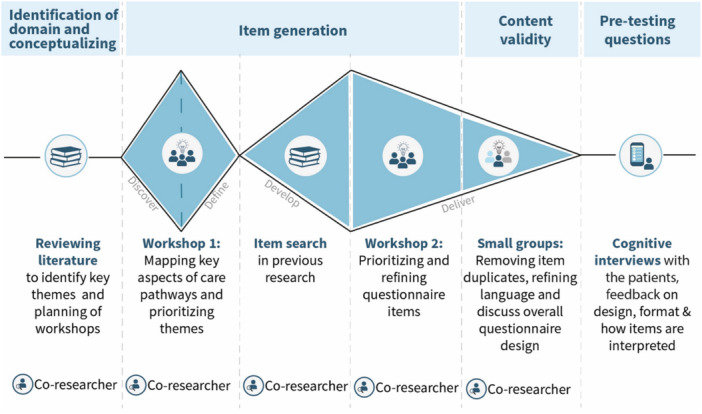
Visual representation of the overall methodology.

The overall methodology for this study followed the initial phases of developing a questionnaire: identification of domain and item generation, content validity, and pre‐testing of questions [12]. A co‐design approach was integral to the entire process, with iterative, creative, steps with collaboration with the co‐researcher, co‐designers at every phase [19]. While Boateng et al. [12] offer a robust method for scale development, our team opted to integrate the Double Diamond framework to enhance the participatory and iterative nature of item generation and refinement (Design Council, 2024). Unlike Delphi methods, which rely on structured consensus among experts, Double Diamond supports open exploration and collaborative design, making it particularly suitable for engaging individuals with lived experience in shaping the questionnaire. An overview of the process with identified phases, activities and participants is presented in Table 1. Additionally, a visual representation of the overall methodology can be seen in Figure 1.

### 2.3 Identification of Domain and Conceptualizing

The aim of this phase was threefold: (I) to identify key themes relevant to measuring the domain patient preferences in mental healthcare, (II) to ensure that no other existing instrument in the literature could be adopted, and (III) to plan the subsequent phases in the overall questionnaire design process.

This phase involved the primary researcher and the co‐researcher. To establish a strong foundation for this collaboration, several meetings were held early in the process to build trust, align goals, and plan the study. These discussions were essential in fostering a sustainable partnership that supported meaningful collaboration and ensured decision to be made collaboratively throughout the project.

A previously conducted scoping review [9] was revisited by the primary researcher to identify key themes in the literature relevant to the measurement of patient preferences. These themes were then critically examined and discussed collaboratively by the primary researcher and the co‐researcher to incorporate early input from people with lived experience and ensure the relevance and applicability of the identified themes.

During this first phase, participants for the subsequent workshops were recruited. Recruitment for workshops is widely recognized as a challenge, requiring a strategic approach to ensure diverse representation across diagnoses, age groups, and genders. To address this, the primary researcher and the co‐researcher collaborated on a comprehensive recruitment strategy, utilizing multiple networks and targeted outreach.

The themes identified during this step informed the design of the workshops purposed for item generation. This ensured that participants had a structured starting point for contributing their perspectives and supported early reflections on face and content validity.

A diverse group in terms of age and gender was successfully recruited for the upcoming workshops.

### 2.4 Item Generation and Material

The aim of the item generation phase was to collaboratively generate questionnaire items that reflect patient preferences in mental healthcare. To achieve the aim, two workshops were conducted in which we employed the Double Diamond framework developed by the British Design Council (Design Council, 2024). In line with the core principle of this framework, the process of item generation was iterative and firstly explored and framed the problem (workshop 1), while secondly focused on developing and delivering solutions (workshop 2 + content validity). All while the co‐researcher and 15 co‐designers were part of this process.

The first workshop was designed to: (I) map out what participants deem important in their care pathways, and (II) to categorize all relevant components of the pathway into themes. In this workshop, we applied the first half of the Double Diamond framework which consists of two steps “discover” and “define.” During the first half of the workshop, a visual elicitation method was used [22]. Participants were asked to select a picture from an assortment of 30 pictures laid out on the table. They used the chosen image to narrate a significant episode from their care pathway, particularly one that had a substantial impact on their recovery. This approach draws on metaphorical thinking to support emotional expression and deeper reflection, often revealing insights that structured questions alone may not uncover. As stories were shared, other participants, along with facilitators, noted down key words identified as essential during the process. Detailed descriptions of these exercises and the workshop timetable can be found in the workshop scripts in Supplementary 1.

In the second half of the first workshop, the collected keywords were utilized to align the stories with existing themes identified in the literature. For instance, when a co‐designer shared a story about feeling heard during consultations with staff, the associated keywords such as communication, being taken seriously, and trust were mapped onto the pre‐existing theme of interactions with healthcare staff. This helped validate the relevance of the literature‐based themes while also capturing the participants lived experiences. If keywords did not correspond to any of the pre‐existing themes, they were set aside for further discussion. Peer‐workers were afterwards encouraged to broaden their perspective, reflecting on the care pathway from the viewpoint of a larger segment of patients they were meeting in their daily work. They were asked to envision future care pathways and consider which elements might become increasingly important over time. Finally, in the end of this first workshop all participants were asked to prioritize the themes in order of importance with green, yellow, and red colors. The use of the discover and define approach, carried out together with co‐designers, ensured that the process started openly and collaboratively. It allowed participants to condense insights collectively, minimizing the need for primary researcher to later “translate” large amounts of qualitative data. Altogether, this exemplifies how the Double Diamond approach supports an open and participatory start to the development process.

In between workshops, the primary researcher returned to the literature and search for items in relevant articles. All findings were discussed with the co‐researcher. This part of the process constitutes the develop step in the second diamond in the Double Diamond framework.

The aim of the second workshop was to select, debate, prioritize, and revise items for the questionnaire. This workshop aligned with the deliver step in the second diamond in the Double Diamond framework, where co‐designers examined, discussed, and rated proposed items. Participants were divided into groups, and each group of participants was assigned 3–4 thematic areas, with corresponding items to evaluate. For example, one thematic area was “physical environment,” with items such as: “single room when you are hospitalized,” “healthy, varied and well‐prepared food during hospitalization,” “good opportunities to have visitors during hospitalization.” The groups were tasked with selecting and discussing these items and making revisions as necessary. A second group critically assessed the decisions from another group allowing for making further revisions if needed.

The two workshops resulted in a comprehensive set of draft items reflecting patient preferences in mental healthcare. These items were further refined and validated during the subsequent content validity phase and contributed to strengthening the questionnaire's face and content validity.

### 2.5 Content Validity

The aim of the content validity phase was to ensure the questionnaire's content was clear, relevant, and comprehensive by incorporating feedback from co‐designers and project collaborators thereby strengthening both face and content validity. This phase consisted of four meetings between the primary researcher, the co‐researcher, a co‐designer, a statistician, and a psychiatric project nurse.

Prior to the first meeting the selected items were structured in Redcap a secure web application for building and managing online surveys [23]. During the meetings, the participants reviewed all the items, dedicated to removing duplicates, refining language to ensure clarity and comprehension and selecting the most appropriate response categories. The psychiatric project nurse ensured that the items were clinically relevant and feasible to use in practice, while the statistician focused on the methodological soundness of the questionnaire, including response formats, item structure and overall design to support later psychometric testing. The questionnaire's overall design was also discussed, with the co‐researcher and the co‐designer providing valuable recommendations for the data collection process. This phase finalized the items and ensured the questionnaire was ready for pre‐testing.

### 2.6 Pre‐Testing Questions

The aim of this phase was to evaluate the questionnaire's usability, language clarity, and overall design through feedback from end‐users. Thirteen co‐designers recruited from outpatient clinics participated.

These co‐designers were invited to finalize the questionnaire in person. This phase consisted of a “thinking aloud method,” which is a method that requires subjects to talk while solving a problem or performing a task [24]. Co‐designers were instructed to read the information page, the consent page, and complete the questionnaire while saying out loud what they think about language, design, length, items, the categories of answer and general understanding. This approach allowed this phase to serve both as a usability test and a cognitive interview stage, providing feedback not only on design and format but also on how items were interpreted. Co‐designers were invited to the test until no further adjustments were suggested. This phase provided actionable insights, ensuring that the final questionnaire was user‐friendly and comprehensible for the target population thus further enhancing face validity.

## 3 Results

The development of the questionnaire was carried out through a four‐phase process. Each phase contributed to shaping the final tool by integrating the perspectives of co‐designers and project collaborators and systematically refining its content and format. The results from each phase are presented in the following sections.

In the first phase, 9 overarching themes were found in the literature to be important for patients, see Table 2 above.

**Table 2:** Overview of the results.

Phase	Output
Identification of domain and conceptualizing	Nine overarching themes were identified:
	1. Interactions with health care staff,
	2. Care and treatment,
	3. Pharmacological treatment,
	4. Therapy,
	5. Physical environment,
	6. Access and time,
	7. Involvement and participation,
	8. Continuity of care,
	9. Information
Item generation	Two additional themes were generated: (10) meaningful community connections and (11) the person as a whole.
	Preliminary pool of 242 items was narrowed down to a set of 58 items.
Content validity	The final questionnaire was reduced to 54 items.
	Recommendation for data collection: Avoiding unnecessary complexity
	All instructions and communication should be written in clear, straightforward language
	Comprehensive yet concise information in advance
	The option to submit their data anonymously
Pre‐testing questions	Several design changes were made, including adding a “Not relevant for me” response option, color‐coding importance and satisfaction columns, and refining language. This phase also helped estimate completion time, informing planning for data collection

In the item generation phase, workshop 1 was organized to provide participants with the opportunity to contribute additional themes that would inform the subsequent item generation. Two additional themes were generated in this workshop: “meaningful community connections” and “the person as a whole.” In terms of meaningful community connections, co‐designers highlighted the importance of being part of a supportive and inclusive social network that fosters a sense of belonging and purpose. They expressed that meaningful communities are not just about having people around but being surrounded by individuals who understand, accept, and value them for who they are. These communities could include family, friends, peer groups, or local organizations. The theme of “the person as a whole” reflected the patients' desire to be recognized beyond their diagnosis or symptoms. They expressed a need for care providers and others to view them as individuals with unique histories, strengths, and needs, rather than solely focusing on their mental illness.

In the second workshop, participants were divided into small groups and tasked with reviewing a preliminary pool of 242 items. The groups engaged in detailed discussions to evaluate each item's relevance, clarity, and applicability to capturing patient preferences. Through this collaborative process, they systematically identified redundancies, refined ambiguous phrasing, and prioritized items based on their perceived importance and utility. This iterative and focused effort resulted in a significant reduction of the item pool, narrowing it down to a refined set of 58 items.

In the content validity phase, the final questionnaire was reduced to 54 items. The final questionnaire is provided in the supplementary 1. In this phase recommendation for data collection was made. Firstly, the co‐researcher and the co‐designer pointed towards avoiding unnecessary complexity or length of the informational material to respect patients' time and attention. Secondly, from their perspective all instructions and communication should be written in clear, straightforward language that is easy to understand. Patients should be provided with comprehensive yet concise information in advance, enabling them to fully understand the purpose of the tool and the process involved in providing their responses. Finally, patients should have the option to submit their data anonymously to ensures privacy and may result in more accurate and meaningful data collection.

In the last phase several different design changes were made based on co‐designer feedback. Firstly, the response category of “I don't know” was extended to also include “Not relevant for me” and this was elaborated on the information page in the beginning of the questionnaire. Another design change was made due to feedback from a co‐designer. The change was to make the columns of importance and satisfaction in two separate colors. Several minor corrections to language were also made.

We also used this phase to estimate time consumption of filling out the questionnaire. This gave us an approximate timeframe for data collection.

## 4 Discussion

This study presents 54 novel, co‐designed questionnaire items that capture what matters to adult patients in Danish mental healthcare. Unlike traditional tools, our questionnaire was shaped by co‐designers or the co‐researcher at every stage—from theme generation to item refinement—ensuring relevance and resonance. The process surfaced two unique themes not found in prior research: the person as a whole and meaningful community connections. These additions reflect a broader, more human‐centered understanding of care and underscore the value of throughout patient involvement. This study focused on content and face validation of the questionnaire. Further statistical validation, including exploratory factor analysis (EFA) and confirmatory factor analysis (CFA), will be necessary to establish the instrument's construct validity and refine its psychometric properties.

Our approach not only distilled the most critical aspects of patient preferences but also addressed common barriers to comprehension and relevance found in existing measures. Minor design and wording adjustments following the pre‐testing of questions further enhanced usability and feasibility for routine data collection.

In the following discussion, we first critique the strengths and limitations of our co‐design methodology, then evaluate the questionnaire's preliminary psychometric performance, and finally consider the practical implications of integrating person‐centered preference measurement into everyday mental health practice.

The study by [25] which is the most comparable to ours when looking at the methodology and objectives, employed a more traditional three‐stage user‐led Delphi methodology. In this approach, relevant items were first identified through a literature review before being rated by a Service User Reference Group. Although Byrne and Morrison [25] structured their themes differently, organizing them into four categories—“what I most want help with,” “what I want for the long term,” “what I would prefer when receiving mental health help,” and “what I prefer when meeting with mental health staff”—their findings share many similarities with ours. Frequently endorsed treatment preferences in their study included individualized care, collaborative decision‐making, greater access to information and treatment choices, privacy during staff interactions, and age‐appropriate care. Privacy was not explicitly present in our data. This omission could be due to contextual differences in the Danish healthcare setting, where privacy is generally ensured, as most interactions with staff take place in private rooms.

While there is considerable overlap in the kinds of preferences identified, our study surfaced two additional themes that extend beyond the scope of previous work [25, 26, 27]. These themes (“the person as a whole” and “meaningful community connections”) demonstrate how co‐design can expand our understanding of what matters in mental healthcare today. For example, “The person as a whole” reflects patients' desire to be met not solely as recipients of treatment but as full human beings with identities, histories, relationships, and aspirations that stretch far beyond diagnosis or symptom management. Items under this theme such as *“help tailored to your needs and desires”* and *“personnel support for you regarding who you are outside of your illness”* express a wish to be understood holistically. The theme resonates with broader shifts in the field, going from a more traditional biomedical approach to a recovery‐oriented approach [28].

Meaningful community connections, meanwhile, introduced a dimension rarely included in traditional patient experience measures [29]. Co‐designers emphasized the importance of support that extends beyond clinical care, focusing on life outside the hospital and the value of social connections beyond inpatient settings. Participants highlighted that mental health services often lack attention to the transition back into everyday life and the integration into communities that are essential for recovery. They argued that psychiatric treatment shouldn't be viewed as an isolated event but as part of a continuum that includes belonging, contribution, and recognition in everyday life. This perspective resonates with the emerging concept of “mattering,” which holds that people have an existential need both to feel valued by others and to add value to their communities [30, 31]. This emphasis on meaningful community connections also aligns with key priorities in the Danish recommendations for a 10‐year action plan which advocates for a more community‐based approach to mental [32]. By including items that reflect this broader view of a patient's episode of care, the co‐designed questionnaire offers a way to operationalize and monitor patient experiences in line with national policy directions.

Methodologically, these themes (“the person as a whole” and “meaningful community connections”) likely would not have emerged without a truly participatory and iterative process. Cargo and Mercer's (2020) overview of participatory research highlights how iterative, partnership‐based methods—engaging stakeholders in every choice point—are essential for surfacing context‐specific insights that traditional approaches miss [33]. Involving a co‐researcher with lived experience and opening workshops with free‐form dialog revealed perspectives that might otherwise have remained hidden [16]. Rather than imposing a narrowly defined construct, we invited co‐designers to articulate what mattered most to them throughout their episode of care, allowing new themes to emerge organically. By contrast, traditional methods—such as the Delphi technique or pre‐structured focus groups—risk silencing these insights through rigid, predefined methods [34].

While our open, participatory approach yielded rich insights, it also introduced potential biases. Workshop dynamics may have allowed more assertive participants to dominate discussions and inadvertently marginalize quieter voices [35, 36]. Unlike the anonymity of traditional Delphi panels our reliance on group‐based prioritization may have skewed item selection toward the preferences of those most comfortable engaging. Moreover, despite targeted recruitment efforts, we were unable to secure adequate representation from ethnically diverse groups, limiting the broader applicability of our findings and highlighting the imperative for future research to foreground cultural diversity explicitly [37].

Another limitation relates to the initial development of the item pool. While the co‐design process emphasized participatory methods, the first draft of questionnaire items was derived primarily from a review. This approach was chosen to ensure that the questionnaire built on existing evidence, offering a comprehensive foundation for discussion. However, it also introduced a researcher‐driven perspective that may have influenced the direction and framing of the items. An alternative approach where participants had generated items might have yielded different formulations. To counterbalance the initial researcher‐led item pool, we built in iterative feedback loops where participants could challenge, revise, and reshape the questionnaire. This ensured the final instrument speaks the language of lived experience—not just academic theory.

## 5 Conclusion

This study demonstrates how co‐design can strengthen traditional questionnaire development by ensuring that resulting instruments are both valid and meaningful to patients. These findings show how co‐design broadens the understanding of what matters in mental healthcare, extending beyond clinical encounters to encompass identity, belonging, and everyday life. The resulting 54 questionnaire items offer a strong foundation for further validation, with hopes of a final metric being able to guide person‐centered mental health services in Denmark.

## Author Contributions

**Klaudia Kristensen:** conceptualization, data collection, formal analysis, project administration, writing – original draft, writing – review and editing. **Anna Skov:** conceptualization, data collection, formal analysis. **Solvejg Kristensen:** writing – original draft, writing – review and editing, supervision. **Ottar Ness:** writing – review and editing, supervision. **Søren Paaske Johnsen:** conceptualization, supervision. **Jan Mainz:** conceptualization, supervision. **Malene Terp:** conceptualization, data collection, project administration, writing – original draft, writing – review and editing, supervision.

## Funding

The authors received no specific funding for this work.

## Conflicts of Interest

The authors declare no conflicts of interest.

## Supporting information

Supplementary Information

## Data Availability

The data that support the findings of this study are available from the corresponding author upon reasonable request. Due to the qualitative nature of the co‐design process, full transcripts and raw data are not publicly available to protect the confidentiality of co‐designers.
