# Supplementary material for: Initial Co‐Design Development of a Questionnaire to Measure Patient Preferences in a Danish Mental Healthcare Setting
Source: Health Expect. 2026 Jan 15;29(1):e70561. doi: 10.1111/hex.70561 (PMC12808807; doi:10.1111/hex.70561)
Supplement: Supplementary file 1 — Supplementary Information [file HEX-29-e70561-s001.docx]

**Supplementary**

### **Finalized questionnaire**

Thank you for taking the time to help us improve our services.

The form you are about to fill in has been developed in collaboration with both patients and healthcare professionals. It is part of a larger project that will give us comprehensive insight into what patients who use psychiatric services find important.

Thus, there is no right or wrong answer. We just want to hear your honest opinion. The form consists of 11 themes, which are presented randomly.

You must first rate how important a statement is for a successful course of treatment on a scale from 1-5.  At the same time, you must rate how satisfied you are with the same statement on a scale from 1-5.

At the end of each page, you must additionally decide which statement is the most important.

There is an option to answer *not relevant* if the question does not make sense to you or you have not received treatment that would allow you to properly address the statement.

Feel free to take your current course of treatment in Psychiatry as a starting point.

—----------------------------------------------------------------------------------------------------------------------------------------

**Care and Treatment**

In this section, we would like to know how important the following statements about care and treatment are for you to experience a good psychiatry course. We would also like to know how satisfied you are with your experience and ask that you answer statements about your course of treatment. Feel free to take your own treatment course as a starting point.

The Importance and Satisfaction with Care and Treatment

| Item | Importance  (not important at all, not important, neutral, important, very important) + not relevant | Satisfaction  (not satisfied at all, not satisfied, neutral, satisfied, very satisfied) |
| --- | --- | --- |
| 1. Improvement of your symptoms | 1,2,3,4,5 + 6 | 1,2,3,4,5 + 6 |
| 1. Treating the cause of your symptoms | 1,2,3,4,5 + 6 | 1,2,3,4,5 + 6 |
| 1. Clarity and understanding of diagnosis | 1,2,3,4,5 + 6 | 1,2,3,4,5 + 6 |
| 1. Treatment of physical illness during your course of treatment | 1,2,3,4,5 + 6 | 1,2,3,4,5 + 6 |
| 1. Treatment of addiction | 1,2,3,4,5 + 6 | 1,2,3,4,5 + 6 |
| 1. Open dialogue about suicidal thoughts | 1,2,3,4,5 + 6 | 1,2,3,4,5 + 6 |
| 1. Learning about co-responsibility in your recovery | 1,2,3,4,5 + 6 | 1,2,3,4,5 + 6 |
| 1. Help from employees with a patient background (peers) | 1,2,3,4,5 + 6 | 1,2,3,4,5 + 6 |

If you had to choose only one of the above statements as the most important, which would it be?

1 2 3 4 5 6 7 8

**Interactions with health care staff**

In this section, we would like to know how important the following statements about meeting with the personnel are for you to experience a good course of treatment in psychiatry. We would also like to know how satisfied you are with your experience regarding the following statements in relation to your course of treatment. Please take your own treatment course as a starting point.

The Importance and Satisfaction with

| Item | Importance  (not important at all, not important, neutral, important, very important) + not relevant | Satisfaction  (not satisfied at all, not satisfied, neutral, satisfied, very satisfied) |
| --- | --- | --- |
| 1. Trustful cooperation with staff | 1,2,3,4,5 + 6 | 1,2,3,4,5 + 6 |
| 1. Empathy and understanding from personnel | 1,2,3,4,5 + 6 | 1,2,3,4,5 + 6 |
| 1. Curiosity and openness from staff | 1,2,3,4,5 + 6 | 1,2,3,4,5 + 6 |
| 1. Unbiased personnel | 1,2,3,4,5 + 6 | 1,2,3,4,5 + 6 |
| 1. Personnel trust what you say | 1,2,3,4,5 + 6 | 1,2,3,4,5 + 6 |

If you had to choose only one of the above statements as the most important, which would it be?

1 2 3 4 5

**Therapy**

In this section, we would like to know how important the following statements about therapy are for you to experience a good course of treatment in psychiatry. We would also like to know how satisfied you are with your experience regarding these statements in relation to your course of treatment. Please use your own treatment course as a starting point.

The Importance and Satisfaction with

| Item | Importance  (not important at all, not important, neutral, important, very important) + not relevant | Satisfaction  (not satisfied at all, not satisfied, neutral, satisfied, very satisfied) |
| --- | --- | --- |
| 1. Individual therapy | 1,2,3,4,5 + 6 | 1,2,3,4,5 + 6 |
| 1. Group Therapy | 1,2,3,4,5 + 6 | 1,2,3,4,5 + 6 |
| 1. Conversations where trauma is discussed with your clinician | 1,2,3,4,5 + 6 | 1,2,3,4,5 + 6 |
| 1. Therapy amount tailored to your needs | 1,2,3,4,5 + 6 | 1,2,3,4,5 + 6 |
| 1. The knowledge you gain about your illness through education (psychoeducation) | 1,2,3,4,5 + 6 | 1,2,3,4,5 + 6 |

If you had to choose only one of the above statements as the most important, which would it be?

1 2 3 4 5

**Pharmacological treatment**

In this section, we would like to know how important the following statements about pharmacological treatment are for you to experience a good course of treatment in psychiatry.  We would also like to know how satisfied you are with your experience regarding these statements in relation to your course of treatment. Please use your own treatment course as a starting point.

The Importance and Satisfaction with

| Item | Importance  (not important at all, not important, neutral, important, very important) + not relevant | Satisfaction  (not satisfied at all, not satisfied, neutral, satisfied, very satisfied) |
| --- | --- | --- |
| 1. Medical treatment combined with therapy | 1,2,3,4,5 + 6 | 1,2,3,4,5 + 6 |
| 1. Non-medical treatment | 1,2,3,4,5 + 6 | 1,2,3,4,5 + 6 |
| 1. Advantages and disadvantages of medication being discussed with you | 1,2,3,4,5 + 6 | 1,2,3,4,5 + 6 |
| 1. Adjustment to or phasing out of medication being discussed with you | 1,2,3,4,5 + 6 | 1,2,3,4,5 + 6 |
| 1. The right medication is regularly found and adjusted in collaboration with you | 1,2,3,4,5 + 6 | 1,2,3,4,5 + 6 |
| 1. Help managing both psychological and physical side effects | 1,2,3,4,5 + 6 | 1,2,3,4,5 + 6 |

If you had to choose only one of the above statements as the most important, which would it be?

1 2 3 4 5 6

**Continuity of care**

In this section, we would like to know how important the following statements about continuity of care are for you to experience a good course of treatment in psychiatry. We would also like to know how satisfied you are with your experience regarding these statements in relation to your course of treatment. Please use your own treatment course as a starting point.

The Importance and Satisfaction with

| Item | Importance  (not important at all, not important, neutral, important, very important) + not relevant | Satisfaction  (not satisfied at all, not satisfied, neutral, satisfied, very satisfied) |
| --- | --- | --- |
| 1. There is a clear division of responsibilities between you and your clinicians | 1,2,3,4,5 + 6 | 1,2,3,4,5 + 6 |
| 1. Follow-up on treatment after discharge | 1,2,3,4,5 + 6 | 1,2,3,4,5 + 6 |
| 1. Important knowledge being passed between outpatient clinics and inpatient | 1,2,3,4,5 + 6 | 1,2,3,4,5 + 6 |
| 1. Collaboration between the Psychiatry Department, municipality, and personal physician | 1,2,3,4,5 + 6 | 1,2,3,4,5 + 6 |

If you had to choose only one of the above statements as the most important, which would it be?

1 2 3 4

**Involvement and participation**

In this section, we would like to know how important the following statements about involvement and participation are for you to experience a good course of treatment in psychiatry. We would also like to know how satisfied you are with your experience regarding these statements in relation to your course of treatment. Please use your own treatment course as a starting point.

The Importance and Satisfaction with

| Items | Importance  (not important at all, not important, neutral, important, very important) + not relevant | Satisfaction  (not satisfied at all, not satisfied, neutral, satisfied, very satisfied) |
| --- | --- | --- |
| 1. Preparation of treatment plan done in collaboration with you | 1,2,3,4,5 + 6 | 1,2,3,4,5 + 6 |
| 1. Possibility to participate in all meetings regarding your treatment | 1,2,3,4,5 + 6 | 1,2,3,4,5 + 6 |
| 1. Involvement in the prevention of incidents that require force | 1,2,3,4,5 + 6 | 1,2,3,4,5 + 6 |
| 1. Relatives are involved in your course | 1,2,3,4,5 + 6 | 1,2,3,4,5 + 6 |

If you had to choose only one of the above statements as the most important, which would it be?

1 2 3 4

**Access and time**

In this section, we would like to know how important the following statements about access and time are for you to experience a good course of treatment in psychiatry. We would also like to know how satisfied you are with your experience regarding these statements in relation to your course of treatment. Please use your own treatment course as a starting point.

The Importance and Satisfaction with

| Item | Importance  (not important at all, not important, neutral, important, very important) + not relevant | Satisfaction  (not satisfied at all, not satisfied, neutral, satisfied, very satisfied) |
| --- | --- | --- |
| 1. Possibility to book appointments online | 1,2,3,4,5 + 6 | 1,2,3,4,5 + 6 |
| 1. Help when you ask for it | 1,2,3,4,5 + 6 | 1,2,3,4,5 + 6 |
| 1. Personnel have enough time when you meet | 1,2,3,4,5 + 6 | 1,2,3,4,5 + 6 |
| 1. Outpatient treatment available after 3 PM | 1,2,3,4,5 + 6 | 1,2,3,4,5 + 6 |
| 1. Possibility for video consultations | 1,2,3,4,5 + 6 | 1,2,3,4,5 + 6 |

If you had to choose only one of the above statements as the most important, which would it be?

1 2 3 4 5

**The person as a whole**

In this section, we would like to know how important the following statements about the person as a whole are for you to experience a good course of treatment in Psychiatry. We would also like to know how satisfied you are with your experience regarding these statements in relation to your course of treatment. Please use your own treatment course as a starting point.

The Importance and Satisfaction with

| Item | Importance  (not important at all, not important, neutral, important, very important) + not relevant | Satisfaction  (not satisfied at all, not satisfied, neutral, satisfied, very satisfied) |
| --- | --- | --- |
| 1. Personnel support what is meaningful for you | 1,2,3,4,5 + 6 | 1,2,3,4,5 + 6 |
| 1. Help tailored to your needs and desires | 1,2,3,4,5 + 6 | 1,2,3,4,5 + 6 |
| 1. Personnel support for you regarding who you are outside of your illness | 1,2,3,4,5 + 6 | 1,2,3,4,5 + 6 |
| 1. Personnel ask about your past, present and future | 1,2,3,4,5 + 6 | 1,2,3,4,5 + 6 |

If you had to choose only one of the statements as the most important, which would it be?

1 2 3 4

**Meaningful community connections**

In this section, we would like to know how important the following statements about meaningful community [connections] are for you to experience a good course of treatment in psychiatry. We would also like to know how satisfied you are with your experience regarding these statements in relation to your course of treatment. Please use your own treatment course as a starting point.

 The Importance and Satisfaction with

| Item | Importance  (not important at all, not important, neutral, important, very important) + not relevant | Satisfaction  (not satisfied at all, not satisfied, neutral, satisfied, very satisfied) |
| --- | --- | --- |
| 1. Help joining meaningful communities outside of psychiatry | 1,2,3,4,5 + 6 | 1,2,3,4,5 + 6 |
| 1. Networks that include other patients like you | 1,2,3,4,5 + 6 | 1,2,3,4,5 + 6 |
| 1. Experiences from your illness are used in relevant communities | 1,2,3,4,5 + 6 | 1,2,3,4,5 + 6 |

If you had to choose only one of the above statements as the most important, which would it be?

1 2 3 4

**Information**

In this section, we would like to know how important the following statements about information are for you to experience a good course of treatment in psychiatry. We would also like to know how satisfied you are with your experience regarding these statements in relation to your course of treatment. Please use your own treatment course as a starting point.

The Importance and Satisfaction with

| Item | Importance  (not important at all, not important, neutral, important, very important) + not relevant | Satisfaction  (not satisfied at all, not satisfied, neutral, satisfied, very satisfied) |
| --- | --- | --- |
| 1. Easily understood information regarding your treatment | 1,2,3,4,5 + 6 | 1,2,3,4,5 + 6 |
| 1. Information about the possibility of recovering from a mental illness | 1,2,3,4,5 + 6 | 1,2,3,4,5 + 6 |
| 1. Written information about the individual diagnoses | 1,2,3,4,5 + 6 | 1,2,3,4,5 + 6 |
| 1. Information about relevant services outside of Psychiatry (e.g., from municipalities and voluntary organizations) | 1,2,3,4,5 + 6 | 1,2,3,4,5 + 6 |

If you had to choose only one of the above statements as the most important, which would it be?

1 2 3 4

**Physical Environment**

In this section, we would like to know how important the following statements about information are for you to experience a good course in psychiatry. We would also like to know how satisfied you are with your experience regarding these statements in relation to your course of treatment. Please use your own treatment course as a starting point.

 The Importance and Satisfaction with

| Item | Importance  (not important at all, not important, neutral, important, very important) + not relevant | Satisfaction  (not satisfied at all, not satisfied, neutral, satisfied, very satisfied) |
| --- | --- | --- |
| 1. Single Room when you are hospitalized | 1,2,3,4,5 + 6 | 1,2,3,4,5 + 6 |
| 1. Healthy, varied and well-prepared food during hospitalization | 1,2,3,4,5 + 6 | 1,2,3,4,5 + 6 |
| 1. Good opportunities to have visitors during hospitalization | 1,2,3,4,5 + 6 | 1,2,3,4,5 + 6 |
| 1. Homelike decor | 1,2,3,4,5 + 6 | 1,2,3,4,5 + 6 |
| 1. Activities during hospitalization (e.g., sensory room, creative or physical activities) | 1,2,3,4,5 + 6 | 1,2,3,4,5 + 6 |
| 1. Clear signage in the Psychiatry Department | 1,2,3,4,5 + 6 | 1,2,3,4,5 + 6 |

If you had to choose only one of the above statements as the most important, which would it be?

1 2 3 4 5 6

**Workshop scripts**Workshop 1

| **Time** | **Activity** | **Facilitator** | **Materials** |
| --- | --- | --- | --- |
| **09:00–09:10** | **Welcome and introduction to the day** Purpose and outcomes: The day will be used to uncover what is important in a patient's journey in Psychiatry. Additionally, the day will serve as a foundation for Workshop 2, where we will expand on creating a questionnaire | AS & KK | Name tags, breakfast pastries |
| **09:10–09:25** | **Icebreaker: Welcome circle** A ball is thrown around in the circle. When a participant catches the ball, they are asked to state their name and how they feel about being present for this workshop | AS | Ball |
| **09:30–10:15** | **Picture exercise**  Participants are asked to choose a picture which represents a significant episode from their care pathway, particularly one that had a substantial impact on their recovery. They are asked to share the story with a group | KK | Pictures, post-its |
|  | Table facilitators note 3 keywords per story. | Table facilitators |  |
| **10:15–10:25** | **Break** | — | Coffee/tea, snacks |
| **10:25–11:30** | **“Bingo board flip game”** Group work with the keywords from before the break. The bingo board on the table has categories from the literature. The group must match keywords with the categories. | AS | Bingo board design,  post-its |
|  | Table dialogue: Are there any categories which the board does not include? | Table facilitators |  |
| **11:30–12:15** | **Lunch** | — | Lunch, coffee/tea, snacks |
| **12:15–12:45** | **What else is important?**  Participants were encouraged to broaden their perspective, reflecting on the care pathway from the viewpoint of a larger segment of the population. | KK & AS | Reflection template |
| **12:45–13:00** | **Group discussions**  Participants were asked to envision future care pathways and consider which elements might become increasingly important over time | Table facilitators |  |
| **13:00–13:30** | **Prioritization of categories**  What should be emphasized or reduced? | KK | Large design Layout on floor, red markers |
| **13:30–13:45** | **Plenary summary**  Reflections from the prioritization process | AS & KK | — |
| **13:45–14:00** | **Conclusion**  How did it go? What’s next? Any questions? | Anna | — |

**Workshop 2**

| **Time** | **Activity** | **Facilitator(s)** | **Materials** |
| --- | --- | --- | --- |
| **09:00–09:10** | **Welcome and Program** Welcome and intro to the day:  Recap of last workshop, incl. category work Clarify the day’s purpose, roles, and perspectives. | AS & KK | Name tags, board with summary from the last session |
| **09:10–09:20** | **Icebreaker: Check-in with pictures**  Participants were asked to choose a picture and share a current dream (big or small). | AS | Reuse pictures from last session |
| **09:20–10:05** | **Introduction to Exercise** Groups of 2–3 begin with 5 statements per category (2 at a time), then agree on 6 statement Participants can rephrase/suggest new ones. | KK | Small boards for pairs, large boards per category, markers |
| **10:05–10:15** | **Break** | — | Coffee/tea, snacks |
| **10:15–11:45** | **Continue exercise with statements** Continue working on statements | Table facilitators | Same as above |
| **11:45–12:30** | **Lunch** | — | Lunch, coffee, tea, snacks |
| **12:30–12:35** | **Short recap of morning session** | KK | — |
| **12:35–13:15** | **Continue exercise with statements** Continue working on statements | Table facilitators | Same as above |
| **13:15–13:25** | **Break** | — | Coffee/tea, snacks |
| **13:25–13:45** | **Group Discussion: prioritization**  Groups pass statements to the next group The next group vote and make suggestions on post-its. | KK | Post-its for voting |
| **13:45–14:00** | **Conclusion** Wrap-up and next steps Ask for permission to follow up and share results Express a big thank you | AS | — |
